# Sex chromosome evolution in snakes inferred from divergence patterns of two gametologous genes and chromosome distribution of sex chromosome-linked repetitive sequences

**DOI:** 10.1186/s40851-016-0056-1

**Published:** 2016-08-26

**Authors:** Kazumi Matsubara, Chizuko Nishida, Yoichi Matsuda, Yoshinori Kumazawa

**Affiliations:** 1 Department of Information and Basic Science and Research Center for Biological Diversity, Graduate School of Natural Sciences, Nagoya City University, 1 Yamanohata, Mizuho-cho, Mizuho-ku, Nagoya, Aichi 467-8501 Japan; 2 Laboratory of Animal Genetics, Department of Applied Molecular Biosciences, Graduate School of Bioagricultural Sciences, Nagoya University, Furo-cho, Chikusa-ku, Nagoya, Aichi 464-8601 Japan; 3 Department of Biological Science, Faculty of Science, Hokkaido University, North 10 West 8, Kita-ku, Sapporo, Hokkaido 060-0810 Japan; 4 Avian Bioscience Research Center, Graduate School of Bioagricultural Sciences, Nagoya University, Furo-cho, Chikusa-ku, Nagoya, Aichi 464-8601 Japan; 5 Current affiliation: Research Center for Bioinformatics and Biosciences, National Research Institute of Fisheries Science, Japan Fisheries Research and Education Agency, Yokohama, Kanagawa 236-8648 Japan

**Keywords:** Snake, Z chromosome, W chromosome, Phylogeny, Evolution, Gametolog, Repetitive sequences, Heterochromatin

## Abstract

**Background:**

The discovery of differentially organized sex chromosome systems suggests that heteromorphic sex chromosomes evolved from a pair of homologous chromosomes. Whereas karyotypes are highly conserved in alethinophidian snakes, the degeneration status of the W chromosomes varies among species. The Z and W chromosomes are morphologically homomorphic in henophidian species, whereas in snakes belonging to caenophidian families the W chromosomes are highly degenerated. Snakes therefore are excellent animal models in which to study sex chromosome evolution. Herein, we investigated the differentiation processes for snake sex chromosomes using both coding and repetitive sequences. We analyzed phylogenetic relationships of *CTNNB1* and *WAC* genes, localized to the centromeric and telomeric regions, respectively, of the long arms on snake sex chromosomes, and chromosome distribution of sex chromosome-linked repetitive sequences in several henophidian and caenophidian species.

**Results:**

Partial or full-length coding sequences of *CTNNB1* and *WAC* were identified for Z homologs of henophidian species from Tropidophiidae, Boidae, Cylindrophiidae, Xenopeltidae, and Pythonidae, and for Z and W homologs of caenophidian species from Acrochordidae, Viperidae, Elapidae, and Colubridae. Female-specific sequences for the two genes were not found in the henophidian (boid and pythonid) species examined. Phylogenetic trees constructed using each gene showed that the Z and W homologs of the caenophidian species cluster separately. The repetitive sequence isolated from the W chromosome heterochromatin of the colubrid *Elaphe quadrivirgata* and a microsatellite motif (AGAT)_8_ were strongly hybridized with W chromosomes of the viperid and colubrid species examined.

**Conclusion:**

Our phylogenetic analyses suggest that the cessation of recombination between the Z and W homologs of *CTNNB1* and *WAC* predated the diversification of the caenophidian families. As the repetitive sequences on the W chromosomes were shared among viperid and colubrid species, heterochromatinization of the proto-W chromosome appears to have occurred before the splitting of these two groups. These results collectively suggest that differentiation of the proto-Z and proto-W chromosomes extended to wide regions on the sex chromosomes in the common ancestor of caenophidian families during a relatively short period.

**Electronic supplementary material:**

The online version of this article (doi:10.1186/s40851-016-0056-1) contains supplementary material, which is available to authorized users.

## Background

The discovery of differentially organized sex chromosome systems suggests that heteromorphic sex chromosomes evolved from a pair of homologous chromosomes [1, 2]. The first step is thought to have been the acquisition of a novel sex-determining gene on one member of an autosomal pair, followed by accumulation of alleles conferring an advantage to that sex [3, 4]. Meiotic recombination between the proto-sex chromosomes could have been suppressed around the heterologous region to preserve the linkage of these sexually antagonistic genes. Such suppression may have been accelerated by structural changes in chromosomes (e.g., inversion). Suppression of recombination between the sex chromosomes then favored the accumulation of repetitive DNA sequences on the non-recombining regions, increasing the extent of differentiation between sex chromosomes [5].

**Fig. 1:**
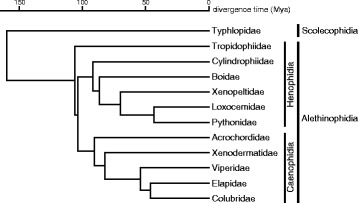
Phylogenetic relationships between snake families. Phylogeny, divergence time and classification are based on Vidal et al. [63], Pyron et al. [49], and Uetz and Hošek [65]

Extant snake species belonging to Serpentes are grouped into two infraorders, Scolecophidia and Alethinophidia. Blind snakes and thread snakes belong to the former and all other snakes belong to the latter (Fig. 1). Alethinophidian species are divided into two superfamilies, Henophidia and Caenophidia (Fig. 1). Snake karyotypes are highly conserved, and most alethinophidian species have a common karyotype whose diploid number is 36, consisting of eight pairs of macrochromosomes and ten pairs of microchromosomes [6–8]. The sex determination system is also conserved in snakes. Nearly all alethinophidian species have ZZ/ZW-type sex chromosomes, in which males have a homomorphic ZZ sex chromosome and females have a heteromorphic ZW. The Z chromosomes are the fourth or fifth largest metacentric chromosomes for most species. In contrast to the highly conserved Z chromosomes, the degeneration status of W chromosomes varies among species [2, 6, 9, 10]. The Z and W chromosomes are homomorphic in the boids and pythonids. Conversely, W chromosomes are highly degenerated and heterochromatic in the colubrids, elapids, and viperids. This characteristic makes snakes good model species for the study of sex chromosome evolution.

We previously constructed, using fluorescence *in situ* hybridization (FISH), a cytogenetic map of the Japanese four-striped rat snake (*Elaphe quadrivirgata*) with more than 180 cDNA clones and found that three genes, *CTNNB1*, *RAB5A*, and *WAC*, were commonly mapped to Z and W chromosomes of this species [11–13]. We also compared structures of sex chromosomes, by C-banding and comparative mapping of Z-linked genes, among three snakes, *E. quadrivirgata* (Colubridae), *Protobothrops flavoviridis* (formerly *Trimeresurus flavoviridis*, Viperidae), and *Python bivittatus* (Pythonidae). The results revealed that W chromosomes of *E. quadrivirgata* and *P. flavoviridis* were highly degenerated, not only in morphology, but also in DNA sequences [12].

Gametologous genes are homologs located on opposite sex chromosomes, which arose through the lack of recombination and subsequent differentiation of sex chromosomes [14]. Although Y chromosomes of eutherian mammals and W chromosomes of neognathous birds are highly degenerated and extensively heterochromatized, the human Y chromosome still contains more than 27 homologs of X-linked single copy genes and pseudogenes [15] and the chicken (*Gallus gallus*) has the Z and W forms of six gametologous genes [14, 16, 17]. In the process of sex chromosome differentiation, suppression of meiotic recombination between entire or partial regions of opposite sex chromosomes facilitates sequence divergence between gametologs. Thus, the evolutionary process of sex chromosome differentiation can be examined by molecular phylogenetic analyses of the gametologs [17–23]. Ellegren and coworkers estimated the date and process of sex chromosome differentiation in birds by comparing gametologs between and within species [17, 19, 23]. Similar to in birds, the evolutionary process of sex chromosome differentiation has also been identified through comparative analysis of gametologous genes in mammals [18, 21], dioecious plants of genus *Silene* [20, 22], and papaya [24]. Recently, massive genome sequencing and transcriptome analyses identified putative gametologous genes in two snakes, *Thamnophis elegans* (Colubridae) and *Sistrurus miliarius* (Viperidae) [25]. The comparative analysis of gametologous genes revealed completely differentiated sex chromosomes in the two species, which suggests that suppression of recombination between the Z and W homologs began before the divergence of the two lineages.

Y chromosomes of eutherian mammals and W chromosomes of neognathous birds are highly heterochromatic and rich in repetitive sequences. Accumulation of repetitive sequences, such as retrotransposons, microsatellite repeats, and ribosomal DNAs, on sex chromosomes has been reported in many species of animals and plants (e.g., [26–29]). The accumulation of repetitive sequences thus is probably associated with heterochromatinization of sex-specific chromosomes (Y and W chromosomes). Accumulation of Bkm repeats, which contain two microsatellite motifs, (GATA)_n_ and (GACA)_n_, was identified on W chromosomes of several colubrid and elapid snakes [30–33], suggesting that these repeat sequences were amplified on the W chromosomes in the common ancestor of Colubridae and Elapidae. We identified amplification of two repetitive sequence families, EQU-BamHI-4 and EQU-BglI-15, on sex chromosomes of *E. quadrivirgata* ([12], Matsubara K. unpublished data). Whereas the EQU-BamHI-4 was amplified in the telomeric regions of both the Z and W chromosomes, the EQU-BglI-15 was intensively amplified on the W chromosome.

In the present study, we sequenced Z and W homologs of *CTNNB1* and *WAC* genes located on centromeric and telomeric regions, respectively, on Z chromosomes [12] from 16 species representing 10 snake families. We also searched for snake homologs of the genes in international nucleotide sequence databases. We constructed molecular phylogenetic trees using these genes to infer the differentiation process for the Z and W chromosomes of snakes based on the divergence patterns of the two genes. We also conducted FISH mapping of (AGAT)_8_ microsatellite motifs and two repetitive sequence families, EQU-BamHI-4 and EQU-BglI-15, for chromosomes of Colubridae, Viperidae, Boidae, and Pythonidae. Finally, we delineated the evolutionary process of sex chromosomes in snakes.

## Methods

### Animals

Table 1 lists the snake species used for this study. One female *E. quadrivirgata* collected in Mie, Japan, was used for chromosome preparation. We also obtained one male, one female, and eggs from a population bred at the Japan Snake Institute. They were sacrificed to collect tissues for DNA and RNA extraction. All the other species, except for *Typhlops* sp., *I. braminus*, *C. ruffus*, *A. arafurae*, *A. granulatus*, and *L. semicarinatum*, were bred at the Japan Snake Institute, Japan. *I. braminus*, a pair of *P. flavoviridis*, and *L. semicarinatum* were captured at Takarajima, Amami-Oshima, and Okinawajima, Ryukyu Islands, Japan, respectively. DNA samples of *Typhlops* sp., *C. ruffus*, *A. arafurae*, and *A. granulatus* were obtained from collections of our laboratory.

**Table 1:** Snake samples used for this study

Infraoder	Superfamily	Family	Species	Abbrev.	2*n* ^*a*^	No. of used animals
Scolecophidia		Typhlopidae	*Typhlops* sp.	TYP	30 (M: 16, m: 14)^b^	1 unknow sex
			*Indotyphlops braminus*	IBR	42 (M: 21, m: 21)^c^	1 female
Alethinophidia	Henophidia	Tropidophiidae	*Tropidophis haetianus haetianus*	THA	un	1 male
		Boidae	*Boa constrictor*	BCO	36 (M: 16, m: 20)	1 male, 1 female
		Cylindrophiidae	*Cylindrophis ruffus*	CRU	un	1 unknow sex
		Xenopeltidae	*Xenopeltis unicolor*	XUN	36 (M: 16, m: 20)^d^	1 male
		Pythonidae	*Python bivittatus*	PBI	36 (M: 16, m: 20)	1 male, 1 female
			*Python molurus*	PMO	36 (M: 16, m: 20)	1 male, 1 female
	Caenophidia	Acrochordidae	*Acrochordus arafurae*	AAR	36^e^	1 male, 1 female
			*Acrochordus granulatus*	AGR	36 (M: 16, m: 20)^f^	1 male
		Viperidae	*Protobothrops flavoviridis*	PFL	36 (M: 16, m: 20)	2 males, 2 females
			*Gloydius blomhoffii*	GBL	36 (M: 16, m: 20)	1 male, 1 female
			*Bitis arietans arietans*	BAR	36 (M: 16, m: 20)	1 male, 1 female
			*Naja kaouthia*	NKA	38 (M: 16, m: 22)^g^	1 male, 1 female
		Elapidae	*Elaphe quadrivirgata*	EQU	36 (M: 16, m: 20)	1 male, 2 females, embryos
		Colubridae	*Lycodon semicarinatum*	LSE	34 (M: 16, m: 18)^h^	1 male, 1 female
			*Rhabdophis tigrinus tigrinus*	RTI	40 (M: 16, m: 24)	1 male, 1 female

^a^The numbers of macrochromosomes (M) and microchromosomes (m) are shown in parentheses. un, the karyotypes have not been identified yet.

^b^The karyotype was identified in our lab [Matsubara et al., unpublished data]

^c^The karyotypic information is derived from Ota et al. [66]

^d^The karyotypic information is derived from Singh et al. [30], and Cole and Dowling [67]

^e^The karyotypic information is derived from CHROMOREP [68]

^f^The karyotypic information is derived from Sharma and Nakhasi [52, 53]

^g^The karyotypic information is derived from Singh [8] and Ray-Chaudhuri et al. [69]

^h^The karyotypic information is derived from Toriba [70]

### Sequencing of Z and W homologs of *CTNNB1* and *WAC* genes

Genomic DNA was extracted from blood or liver tissue by the phenol-chloroform method described by Sambrook *et al*. [34] or with a DNeasy kit (QIAGEN, Venlo, Netherlands), and used for templates in PCR.

We determined, by primer walking, full-length nucleotide sequences of two *E. quadrivirgata* expressed sequence tag (EST) clones, Eq_aB_009012_N17 (BW999995) and Eq_aB_026_N02 (AU312355), previously identified as homologs of *CTNNB1* and *WAC* genes, respectively [11, 12]. We located positions of the intron/exon boundaries on the sequences of *E. quadrivirgata CTNNB1* and *WAC* homologs in comparison with chicken, green anole (*Anolis carolinensis*), and human homologs, and designed primer pairs to amplify partial exons and flanking introns (see Additional file 1 for primer sequences and Additional file 2a for their locations). PCR was conducted with a SpeedStar HS DNA polymerase (Takara, Kusatsu, Japan) under the following conditions: an initial denaturation at 94 °C for 5 min, followed by 35 cycles of 94 °C for 30 s, 50–65 °C for 30 s, 72 °C for 35 s, and 72 °C for 5 min for a final extension. Annealing temperature was changed depending on primers and target species. The PCR products were electrophoresed on 1–3 % agarose gels, and bands were isolated using a QIAquick Gel Extraction Kit (QIAGEN). Extracted DNA was directly sequenced or subcloned using the pGEM-T Easy Vector System (Promega, Madison, WI, USA). For direct sequencing, the 20–40 ng DNA fragments were labeled with a BigDye Terminator v3.1 Cycle Sequencing Kit (Applied Biosystems - Thermo Fisher Scientific, Waltham, MA, USA) using primer sets for each gene fragment based on the manufacturer’s protocol (Applied Biosystems). Both strands of the labeled products were sequenced using an ABI PRISM3700 DNA Analyzer (Applied Biosystems). The cloned DNA fragments were sequenced with T7 and Sp6 primers.

All species, except for *B. arietans*, were used for sequencing and phylogenetic analyses of the *CTNNB1* genes, whereas *Typhlops* sp., *I. braminus*, *T. haetianus*, *B. constrictor*, *C. ruffus*, *X. unicolor*, *P. bivittatus*, *A. arafurae*, *A. granulatus*, *P. flavoviridis*, *N. kaouthia*, and *E. quadrivirgata* were used for phylogenetic analyses of *WAC* genes.

### Rapid amplification of cDNA ends for *E. quadrivirgata CTNNB1* and *WAC* homologs

Total RNA was extracted from *E. quadrivirgata* fetal gonads using an RNeasy Kit (Qiagen). For rapid amplification of cDNA ends (RACE), cDNA was synthesized with a SMARTer® PCR cDNA Synthesis Kit (Clontech, Mountain View, CA, USA) according to the manufacturer’s protocol with the following modification. We used our own primer, 5′-GGC CAC GCG TCG ACT AGT AC(T)_30_ VN-3′, instead of the manufacturer’s primer for synthesis of the first strand of cDNA. Gene-specific primers were designed based on partial sequences obtained in the previous section (Additional file 1).

### Comparison of sequences of *CTNNB1* and *WAC* genes among tetrapods

The open reading frames (ORFs) in *E. quadrivirgata* Z and W homologs of *CTNNB1* and *WAC* were predicted for the full-length cDNA sequences based on sequence similarities with homologs from humans, chickens, and green anole lizards. Putative full-length coding nucleotide and amino acid sequences of the *CTNNB1* Z and W, and *WAC* Z and W homologs were aligned with the homologs from other tetrapods using Clustal Omega [35] at the European Bioinformatics Institute (EMBL-EBI) website. The nucleotide and amino acid sequences of the two genes from the following species were used for the comparison; *CTNNB1* (XM_003223954) and *WAC* (XM_008112381) from *A. carolinensis* (Iguanidae, Squamata), *CTNNB1* (KF803272) and *WAC* (XM_015421414) from *Gekko japonicus* (Gekkonidae, Squamata), *CTNNB1* (NM_205081) and *WAC* (XM_015282076) from *G. gallus* (Phasianidae, Aves), *CTNNB1* (XM_009687805) and *WAC* (XM_009679627) from *Struthio camelus australis* (Struthionidae, Aves), *CTNNB1* (XM_006258718) and *WAC* (XM_014609006) from *Alligator mississippiensis* (Alligatoridae, Crocodilia), *CTNNB1* (XM_005278593) and *WAC* (XM_005290938) from *Chrysemys picta bellii* (Emydidae, Testudines), *CTNNB1* (NM_001286932) and *WAC* (XM_006124777) from *Pelodiscus sinensis* (Trionychidae, Testudines), *CTNNB1* (NM_001904) and *WAC* (NM_016628) from *Homo sapiens* (Hominidae, Primates, Mammalia), and *CTNNB1* (NM_001016958) and *WAC* (XM_012964589) from *Xenopus (Silurana) tropicalis* (Pipidae, Amphibia).

### Identification of snake *CTNNB1* and *WAC* gene sequences in databases

To obtain long coding sequences of the two genes from several snake species, we searched databases for sequences that exhibited high similarities with the *E. quadrivirgata* homologs. BLASTN searches were conducted on the National Center for Biotechnology Information (NCBI) website against whole genome shotgun sequences of female *P. bivittatus* (Pythonidae, BioProject no. PRJNA61243), male *Ophiophagus hannah* (Elapidae, PRJNA201683), female *Crotalus mitchellii pyrrhus* (Viperidae, PRJNA255393), and female *Vipera berus berus* (Viperidae, PRJNA170536) using the full-length cDNA sequences of *E. quadrivirgata CTNNB1* and *WAC* homologs as queries. The contig sequences that exhibited high similarities with the *E. quadrivirgata* cDNA sequences, which consisted of exons, introns, and flanking regions, were selected for each species (Additional file 3). The boundaries between exons and introns within each contig were manually identified using dot-plot matrices between the cDNA sequences and the contig sequences. Next, the exon sequences were combined and the full-length or near full-length coding sequence was determined for homologs of the *CTNNB1* and *WAC* genes in the four species based on the ORF information from other tetrapods.

Available transcriptomic reads were obtained from the NCBI database for the following samples: female *Boa constrictor* blood (Sequence Read Archive (SRA) No. SRR941236), male *Sistrurus miliarius* liver (SRR941232), *Xenopeltis unicolor* liver (SRR629647), and male *Echis coloratus* brain (SRR1328164) (Viperidae). The reads were trimmed based on quality using the DynamicTrim command (*h* = 30), and those shorter than 20 bp were removed using the LengthSort command in SolexaQA [36]. The screened reads were assembled using Trinity [37]. Transcripts of *CTNNB1* and *WAC* genes from each species were identified by the BLASTN search using the full-length cDNA sequences of *E. quadrivirgata* homologs as queries with BlastStation (TM Software, Arcadia, CA, USA). Full-length or near full-length coding sequences for the four snake species were determined based on the sequence similarities to homologs of *E. quadrivirgata* and other tetrapods. Multiple contigs were identified in search of *B. constrictor*, *X. unicolor*, and *S. miliarius* homologs of the *CTNNB1* gene, and for *B. constrictor* homologs of the *WAC* gene. The variation in contigs was probably caused by the presence of transcript variants and precursor mRNA in the tissues. In these cases, transcripts that showed the highest similarity to the homologs of *E. quadrivirgata* and other tetrapods were selected. Two contigs of *S. miliarius* showed high similarities to the *E. quadrivirgata CTNNB1* gene: one was homologous to two-thirds of the coding region and the other was homologous to the remaining one-third. The two contigs shared a 21-bp overlapping sequence at their ends, and thus, they were assembled and the full-length coding sequence was identified as the *S. miliarius* homolog.

The sequences of two *Thamnophis sirtalis* homologs of *CTNNB1* (XM_014069347, XM_014063622) and *WAC* (XM_014065195) were obtained from the International Nucleotide Sequence Database.

### Phylogenetic analysis of *CTNNB1* and *WAC* genes

Sequence alignment was performed with ClustalW [38] implemented in MEGA ver.6 [39], visually checked, and corrected. Neighbor-joining (NJ) and maximum-likelihood (ML) trees were constructed using PAUP ver.4.0a147 [40] and GARLI 2.0 [41], respectively. The most appropriate models and parameters for construction of phylogenetic trees (Additional file 4) were defined for each alignment based on the Bayesian information criterion (BIC) using the jModelTest [42, 43]. The robustness of trees was assessed by bootstrap resampling with 1000 random replications.

We constructed two kinds of molecular phylogenetic trees for the two genes. One tree was constructed with a long alignment that contained full-length coding sequences of the *E. quadrivirgata* Z and W homologs, coding sequences of homologs for other snakes, and non-snake tetrapods identified from genomic databases and transcriptomic data. The other tree was constructed with a short alignment that covered only the amplified and sequenced region of the genes from various snake families and non-snake squamates. The alignments were constructed for only exon sequences because reliable alignments were not obtained with sequences of introns and untranslated regions (UTR). The alignment lengths were 2370, 588, 1974, and 524 sites for the *CTNNB1* gene in the long alignment, the *CTNNB1* gene in the short alignment, the *WAC* gene in the long alignment, and the *WAC* gene in the short alignment, respectively.

The *CTNNB1* sequences from *A. carolinensis*, *Leiolepis reevesii rubritaeniata* (AB490379, Agamidae, Squamata), *G. japonicus*, *C. p. bellii*, *P. sinensis*, *A. mississippiensis*, *S. c. australis*, *G. gallus*, *H. sapien*s, and *X. tropicalis,* and the *WAC* sequences from *A. carolinensis*, *L. r. rubritaeniata* (AB490381), *C. p. bellii*, *P. sinensis*, *A. mississippiensis*, *Alligator sinensis* (XM_014520047, Alligatoridae, Crocodilia), *S. c. australis*, *G. gallus*, *H. sapien*s, and *X. tropicalis* were used for construction of phylogenetic trees. The sequences of *Pogona vitticeps* (Agamidae, Squamata) homologs of *CTNNB1* and *WAC* genes were obtained from the annotated genome (Pogona pvi1.1) through a genome browser available at https://genomics.canberra.edu.au/gbrowse/gbrowse/pogona_pvi1.1/ [44] and included in the phylogenetic analyses.

### Chromosome preparation and FISH

Chromosome preparation and FISH were performed as described in our previous studies [11, 12, 45–47]. Chromosome slides were made from blood lymphocytes and/or fibroblast cells taken from heart tissues of *B. constrictor*, *P. bivittatus*, *P. flavoviridis*, *B. arietans*, *G. blomhoffii*, *E. quadrivirgata*, and *R. tigrinus*. The DNA clones of the two sex chromosome-specific repetitive elements obtained from *E. quadrivirgata*, EQU-BamHI-4 and EQU-BglI-15 ([12], Matsubara K. unpublished data), were labeled using a nick translation kit (Roche Diagnostics, Basel, Switzerland) with biotin-16-dUTP (Roche Diagnostics). Hybridization was conducted at 37 °C for one day. The slides were reacted with FITC-avidin (Roche Diagnostics), and then stained with propidium iodide (PI). The fluorescein-labeled oligonucleotide of (AGAT)_8_ was purchased from Rikaken (Nagoya, Japan) and used for FISH with the protocol described in our previous studies [46, 47].

## Results

### Sequencing of Z and W homologs of *CTNNB1* and *WAC* genes

PCR using the three primer sets for *CTNNB1* genes (Eq-CTNNB1-11-F × 13-R, Eq-CTNNB1-int12-F × 14-R, and Eq-CTNNB1-14-F × 15-R) gave rise to a band common to both males and females and a female-specific band in *E. quadrivirgata* (e.g., Additional file 2b). DNA fragments purified from these bands were sequenced to confirm they were parts of the *CTNNB1* gene. Thus, the DNA sequences from the common bands and the female-specific bands were identified as *CTNNB1* Z and W homologs, respectively. Similarly, PCR using the two primer sets for *WAC* (Eq-WAC-6-F × 7-R and Eq-WAC-8-F × 9-R) produced a band common to both males and females, and a female-specific band in *E. quadrivirgata* (data not shown). The DNA fragments from all these bands were sequenced to confirm they were parts of the *WAC* gene. Thus, the sequences from the common bands and the female-specific bands were identified as *WAC* Z and W homologs, respectively. Full-length cDNA of *E. quadrivirgata CTNNB1* Z and W homologs and *WAC* Z and W homologs were obtained by the RACE method with specific primers for the Z and W homologs of each gene (Additional file 1), and the sequences were registered with the International Nucleotide Sequence Database Collaboration (INSDC) under the accession numbers shown in Additional file 5.

PCR for the other snake species revealed that all five primer sets described above produced a band common to both males and females and a female-specific band for the acrochordid, viperid, elapid, and colubrid species examined (e.g., Additional file 2c). Sequencing the DNA fragments from these bands also demonstrated they represented Z and W homologs for these species. In contrast, all the five primer sets gave rise to a single band common to males and females for *P. bivittatus* and *B. constrictor* (e.g., Additional file 2c). The DNA fragments purified from these single bands were cloned and at least four clones were sequenced to confirm they were identical among clones and between males and females. Specifically, sex-specific sequences were not found for the two genes in *P. bivittatus* and *B. constrictor.* Only one individual of *Typhlops* sp., *I. braminus*, *T. haetianus*, *C. ruffus*, *X. unicolor*, and *A. granulatus* were used for sequencing the partial *CTNNB1* and *WAC* gene sequences (Table 1). All five primer sets produced a single band for *Typhlops* sp., *T. haetianus*, *C. ruffus*, *X. unicolor*, and *A. granulatus*. In *I. braminus*, although the three *CTNNB1* primer sets and the Eq-WAC-6-F × 7-R primer set produced single bands, the remaining primer set did not provide amplified bands (data not shown). The nucleotide sequences of amplified products were confirmed as partial sequences of the *CTNNB1* and *WAC* genes in all six species. Other primers (snake-WAC-7-F, snake-WAC-8-R, and snake-WAC-W-8-R) were designed using available sequence data to amplify partial sequences from exon 7 to exon 8 (Additional file 1). With these new primers, partial sequences from exon 7 to exon 8 were determined in all species, except for the *P. flavoviridis* Z homolog, *T. haetianus*, and *C. ruffus*. All partial sequences of the two genes obtained in this study have been registered with the INSDC; accession numbers are shown in Additional file 5.

### Comparison of CTNNB1 and WAC sequences among tetrapods

**Fig. 2:**
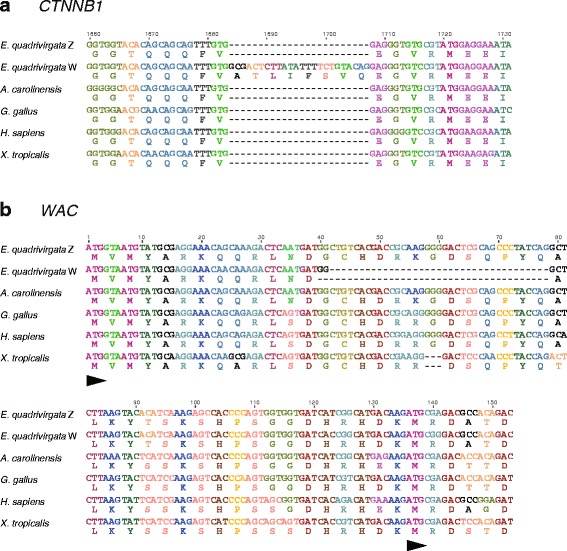
Comparison of partial nucleotide and amino acid sequences of *CTNNB1* and *WAC* genes. Nucleotide and amino acid sequences are aligned between the homologs of *CTNNB1* (**a**) and *WAC* (**b**) genes in five tetrapod species: *E. quadrivirgata*, *A. carolinensis*, *G. gallus*, *H. sapiens* and *X. tropicalis*. Numbers on the alignments indicate nucleotide positions from the translation initiation sites. Arrowheads in **b** indicate two predicted translational initiation sites

Amino acid sequences of the *CTNNB1* genes were highly conserved among the homologs of tetrapod species (Fig. 2a, Additional files 6 and 7). The putative amino acid sequence of the *E. quadrivirgata CTNNB1* Z homolog showed more than 99 % similarities to the homologs of the other amniotes and 97.7 % similarity to the *X. tropicalis* homolog. An insertion (24 bp, 8 aa) was identified at the 1684th site from a start codon in the *E. quadrivirgata CTNNB1* W homolog (Fig. 2a and Additional file 7), and the W homolog exhibited approximately 97 % similarity to the homologs of the other amniotes and 95.3 % similarity to the *X. tropicalis* homolog (Additional file 6).

In contrast to the *CTNNB1* genes, amino acid sequences of *WAC* genes were relatively divergent among the homologs of tetrapod species compared. The amino acid sequence of the *E. quadrivirgata* Z homolog exhibited 92.7 % similarity to the *A. carolinensis* homolog, approximately 91 % similarity to the homologs of the chicken and the painted turtle, 88.7 % similarity to the human homolog, and 82.6 % similarity to the *X. tropicalis* homolog (Additional files 6 and 8). Two ORFs, which corresponded to two chicken transcript variants (variant X1: XM_015282076.1, variant X4: XM_015282080), two human transcript variants (variant X1: NM_016628, variant X2: NM_100264), and two *X. tropicalis* transcript variants (variant X1: XM_012964589, variant X3: XM_012964591), were identified in the putative amino acid sequence of the *E. quadrivirgata WAC* Z homolog (Fig. 2b and Additional file 8). The W homolog showed lower similarities in the amino acid sequence with the homologs of the other tetrapod species (Additional file 6). Furthermore, the W homolog did not retain a longer ORF because of a 37-bp deletion at the 42nd site from a start codon, which would cause a frameshift (Fig. 2b and Additional file 8). Although a shorter ORF starting from the second putative start codon was retained in the W homolog, a few additional deletions specific to the W homolog were also identified in the ORF sequence (Additional file 8).

### Molecular phylogeny of *CTNNB1* and *WAC* genes

**Fig. 3:**
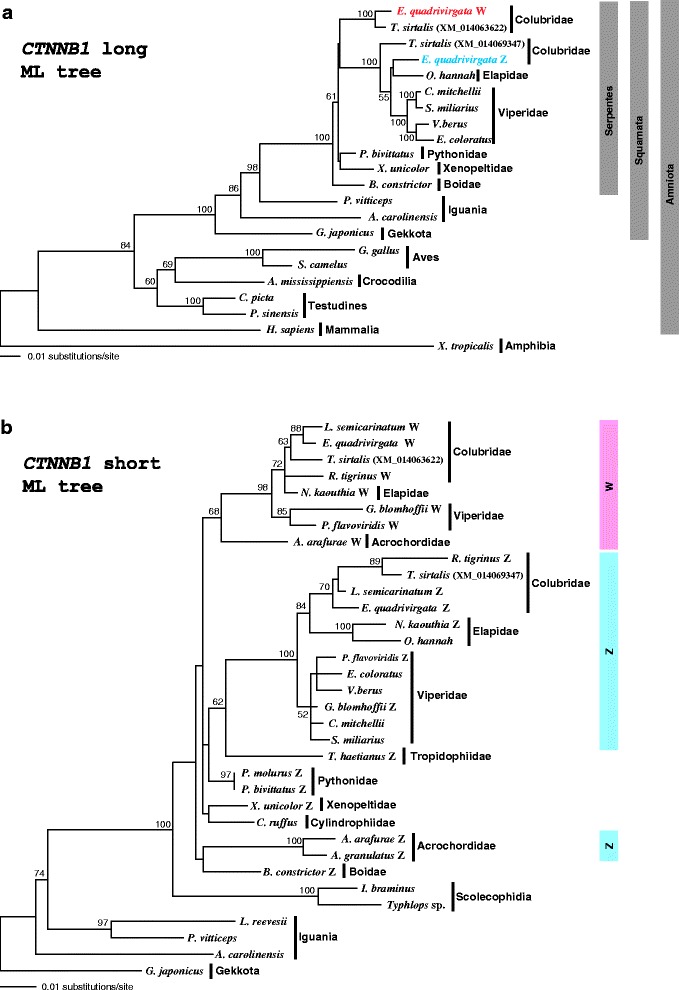
Molecular phylogenetic trees of *CTNNB1* genes. Maximum-likelihood trees of *CTNNB1* genes were constructed with the long alignment for 20 tetrapod species (**a**) and the short alignment for 26 squamate species (**b**). Bootstrap values (>50 %) are shown on each node. Classification is shown on the right side of species. Blue and pink bars in **b** show clades of Z and W homologs of caenophidian species, respectively

**Fig. 4:**
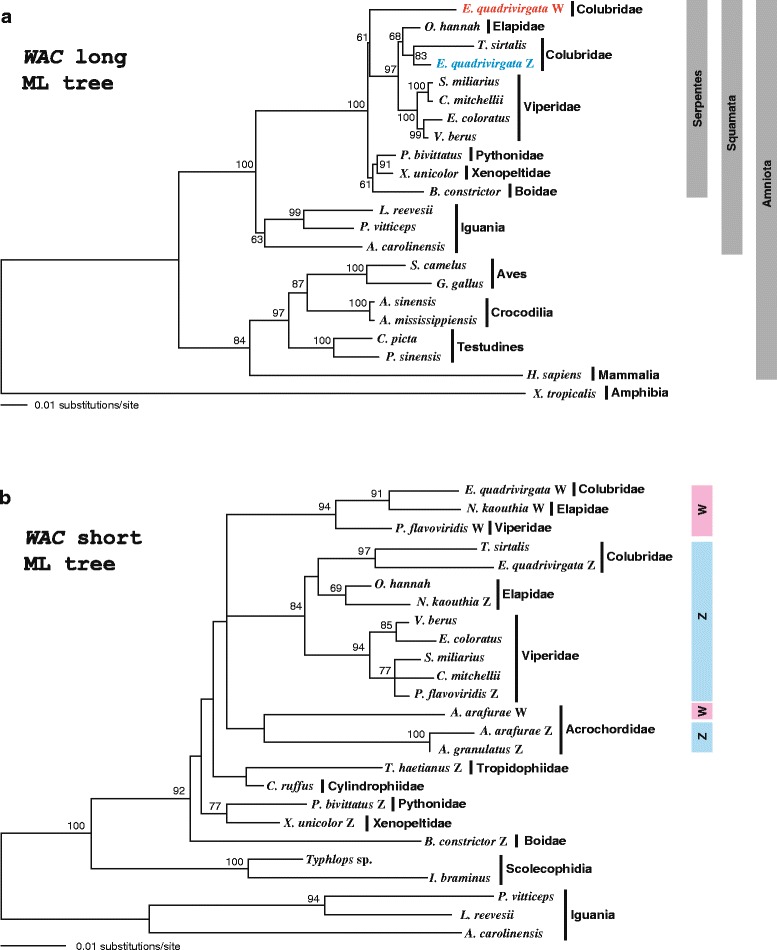
Molecular phylogenetic trees of *WAC* genes. Maximum-likelihood trees of *WAC* genes were constructed with the long alignment for 21 tetrapod species (**a**) and the short alignment for 21 squamate species (**b**). Bootstrap values (> 50 %) are shown on each node. Classification is shown on the right side of species. Blue and pink bars in **b** show clades of Z and W homologs of caenophidian species, respectively

Phylogenetic trees were constructed for each of the *CTNNB1* and *WAC* genes (Figs. 3 and 4 for ML trees and Additional files 9 and 10 for NJ trees). Phylogenetic relationships among amniote species reconstructed using the *CTNNB1* genes from the long alignment (Fig. 3a and Additional file 9a) were in good agreement with other molecular phylogenetic studies [48]. The human homolog was positioned as a sister group to reptiles, and reptiles were divided into two primary clades that corresponded to Archosauromorpha (Testudines, Crocodilia and Aves) and Squamata. Snake species are traditionally divided into three primary groups, Scolecophidia, Henophidia, and Caenophidia (Fig. 1). Although recent molecular studies [49–51] have suggested non-monophyly of the Scolecophidia and Henophidia, they established a clade comprising four henophidian families (Cylindrophiidae, Boidae, Xenopeltidae, and Pythonidae). However, the clustering of henophidian homologs was not conspicuous in the *CTNNB1* tree from the long alignment (Fig. 3a and Additional file 9a). The homolog of *B. constrictor* diverged first from those of the other henophidian and caenophidian species, and thus, the phylogenetic relationships of three henophidian homologs did not completely match the common cladogram shown in Fig. 1. In a caenophidian clade, *E. quadrivirgata* Z homologs clustered with homologs of other caenophidian species. Whereas one *T. sirtalis* homolog (XM_014069347) was included in this cluster, the other *T. sirtalis* homolog (XM_014063622) formed a clade with the *E. quadrivirgata* W homolog (Fig. 3a and Additional file 9a), suggesting that the latter two sequences represented W homologs in Colubridae.

In the *CTNNB1* trees constructed from the short alignment, the second homologs (i.e., W homologs) of eight caenophidian species from Colubridae, Viperidae, Elapidae, and Acrochordidae clearly formed a monophyletic group with 68 and 55 % bootstrap support (Fig. 3b and Additional file 9b). In addition, the first homologs (i.e., Z homologs) of species from three caenophidian families (Colubridae, Viperidae, and Elapidae) also comprised a monophyletic group with strong bootstrap values (100 and 99 %). However, as in the long-alignment trees, homologs of henophidian species from Pythonidae, Xenopeltidae, Cylindrophiidae, and Boidae were not monophyletic. It should be noted that Z homologs of acrochordid species clustered with those of other caenophidian species in the NJ tree, whereas this was not the case in the ML tree, indicating that the phylogenetic position of the acrochordid Z homologs was not resolved well with our dataset.

The *WAC* trees from the long alignment mostly reconstructed the common cladogram of the amniotes [48] and the henophidian snakes (Fig. 1), except for the position of a human homolog, which exhibited a sister-group relationship to Archosauromorpha in ML tree (Fig. 4a and Additional file 10a). The branching patterns of caenophidian homologs were similar to those in the *CTNNB1* trees from the long alignment. The *E. quadrivirgata* W homolog diverged from the caenophidian Z clade after splitting into the caenophidian and henophidian homologs (Fig. 4a). A *T. sirtalis* homolog (XM_014065195) clustered with the *E. quadrivirgata* Z homolog in the ML tree with 83 % bootstrap support (Fig. 4a), whereas it clustered with the *E. quadrivirgata* W homolog in the NJ tree, albeit with a lower (<50 %) bootstrap support (Additional file 10a).

In the *WAC* trees from the short alignment (Fig. 4b and Additional file 10b), homologs of henophidian species from Pythonidae, Xenopeltidae, Cylindrophiidae, and Boidae also did not form a monophyletic clade. Within a caenophidian clade, the Z and W homologs of colubrid, elapid, and viperid species formed mutually monophyletic clades in both ML and NJ trees. The bootstrap values were 84 and 70 % for the Z homolog clades in ML and NJ trees, respectively, and 94 and 83 % for the W homolog clades in ML and NJ trees, respectively. The Z and W homologs of acrochordid species formed a clade separated from the Z and W homologs of the other caenophidian species in the ML tree, although bootstrap support was weak (<50 %) (Fig. 4b). In contrast, in the NJ tree, W homologs of all caenophidian species formed a clade in which the *A. arafurae* W homolog was a sister group to the other species with 72 % bootstrap support and the Z homologs of acrochordid species did not have a sister-group relationship with the Z homolog clade of the other caenophidian species (Additional file 10b). The phylogenetic affiliation of acrochordid Z and W homologs was not therefore conclusive in our trees.

### Comparative FISH mapping of sex chromosome-specific repetitive DNAs

**Fig. 5:**
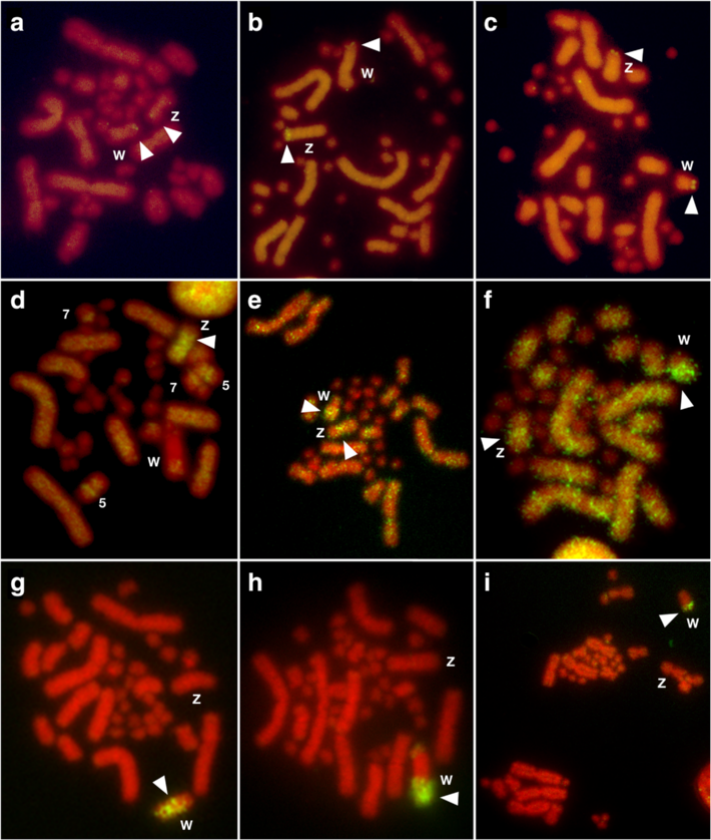
FISH of three repetitive sequences in snakes. FITC-labeled *E. quadrivirgata* BamHI-4 repeat was hybridized to PI-stained metaphase spreads of *B. constrictor* (**a**), *R. tigrinus* (**b**), and *B. arietans* (**c**). *E. quadrivirgata* BglI-15 repeat was hybridized to metaphase spreads of *R. tigrinus* (**d**), *P. flavoviridis* (**e**), and *B. arietans* (**f**). The (AGAT)_8_ microsatellite motif was hybridized to metaphase spreads of *E. quadrivirgata* (**g**), *R. tigrinus* (**h**), and *P. flavoviridis* (**i**). Arrowheads indicate hybridization signals on sex chromosomes

One of two *E. quadrivirgata* repetitive sequences, EQU-BamHI-4 repeat [12], was localized to the distal regions of the short arms of the Z and W chromosomes in all species examined: *B. constrictor* (Fig. 5a), *R. tigrinus* (Fig. 5b), and *B. arietans* (Fig. 5c), as well as in *E. quadrivirgata*, *P. bivittatus,* and *P. flavoviridis* (formerly *Trimeresurus flavoviridis*) [15]. The fluorescent signals were fainter in a henophidian species, *B. constrictor* (Boidae) (Fig. 5a) than in the colubrid *R. tigrinus* and the viperid *B. arietans* (Fig. 5b and c). These variations of signal intensities for this repeat were also observed in a previous FISH experiment [12] in which the repeat showed intense and faint signals in *P. flavoviridis* (Viperidae) and *P. bivittatus* (Pythonidae), respectively, and an intermediate intensity was observed in *E. quadrivirgata* (Colubridae).

Another repetitive sequence, EQU-BglI-15 repeat, was originally identified on the long arm of the W chromosome, the centromeric regions of the Z chromosome, and on three autosome pairs (4th, 6th, and 7th) in *E. quadrivirgata* (Additional file 11a). In *R. tigrinus*, this repetitive sequence provided intense signals on the Z chromosome, the paracentric regions of one pair of small macrochromosomes (5th) and the centromeric regions of the smallest pair of macrochromosomes (7th), but not on the W chromosome (Fig. 5d). The repetitive sequence showed intense fluorescent signals on the long arms of the W chromosomes in *P. flavoviridis* (Fig. 5e), *B. arietans* (Fig. 5f), and *G. blomhoffii* (Additional file 11b), as in *E. quadrivirgata*. A microsatellite motif, (AGAT)_8_, showed intense hybridization signals on the long arm of W chromosomes in *E. quadrivirgata* (Fig. 5g) and *R. tigrinus* (Fig. 5h), and the telomeric regions of the long arm of W chromosomes in *P. flavoviridis* (Fig. 5i) and *G. blomhoffii* (Additional file 11c). In contrast, the EQU-BglI-15 repeat and the microsatellite motif showed no site-specific signals in *B. constrictor* or *P. bivittatus* (data not shown).

## Discussion

### Identification of two gametologous genes in snakes

Except for *E. quadrivirgata* and *P. flavoviridis*, we used only one male and one female, or single individuals of each species, for PCR amplification of partial sequences of *CTNNB1* and *WAC* genes. However, female-specific duplicate amplicons of the two genes were clearly identified in all caenophidian species, including all females of *E. quadrivirgata* and *P. flavoviridis*. Moreover, only single amplicons were recovered from males of all caenophidian species. This indicates that the female-specific amplicons are linked to the caenophidian W chromosomes. Thus, the Z and W homologs of the two genes are differentiated in caenophidian species, including acrochordid species whose Z and W chromosomes have not yet been identified morphologically [52, 53]. The presence of W homologs of the two genes was not identified by FISH in *P. flavoviridis* in our previous study [12]. This might have occurred because of the difficulty of gene mapping in heterochromatic regions.

In two henophidian species, *B. constrictor* and *P. bivittatus*, the female-specific amplicons were not found for the *CTNNB1* and *WAC* genes. Three explanations are possible for this result. The first is that the Z and W homologs are quite similar or not differentiated (i.e., homologous) from each other, an explanation that we favor. The second is that the primers used for PCR did not match the nucleotide sequences of the priming sites on the W homologs of the two genes. The third is that the homologs are already lost from the W chromosomes in henophidian species. However, the third explanation is unlikely because the presence of the W homologs was evidenced by genomic sequencing approaches in *B. constrictor* [28] and by cytogenetic analysis in *P. bivittatus* [15]. Because we used only one individual of the other three henophidian species (*T. haetianus*, *C. ruffus*, and *X. unicolor*), our results do not conclusively reveal whether the sequences for the two genes are identical in the Z and W homologs. Further research is needed for both cytogenetic and genomic characterization of sex chromosomes in henophidian species.

### Evolution of two gametologous genes in snakes

The CTNNB1 protein is necessary for the adhesive function of cadherins, and has a role in mediating the canonical Wnt signaling pathway and regulating gene transcription [54]. Thus, this gene is ubiquitously expressed in tetrapod species, such as humans, mice, chickens, and *X. tropicalis* (NCBI UniGene). In the context of the sex determination pathway, ectopic stabilization of this gene causes phenotypic sex reversal from male to female in laboratory mice [55]. Although the exact function of the WAC protein is unknown, it contains a WW domain, a protein module found in a wide range of signaling proteins [56]. The *WAC* gene is also expressed in many tissues in tetrapod species (NCBI UniGene). Thus, the two genes probably have important functions common among tetrapod species. The snake Z homologs of the two genes, in particular the *CTNNB1* gene, highly conserved amino acid sequences, suggesting that they have evolved under a strong purifying selection and retained the common function. Because the *E. quadrivirgata* W homologs of the two genes have retained complete ORFs, there is no evidence for their existence as pseudogenes. However, the *E. quadrivirgata* W homologs exhibited fewer amino acid sequence similarities with the homologs of the other tetrapod species. Specific indels were also identified in the amino acid sequences of the W homologs. Thus, the *E. quadrivirgata* W homologs of *CTNNB1* and *WAC* genes may have acquired somewhat diverged functions.

The phylogenetic trees of the two gametologs from the long alignments showed that the divergence between Z and W homologs of *E. quadrivirgata* occurred soon after the caenophidian homologs diverged from the henophidian ones (Figs. 3a and 4a; Additional files 9a and 10a). The phylogenetic trees of the two genes from the short alignment, which included more taxa, basically fit this interpretation. The clade of Z homologs of viperid, colubrid, and elapid species and that of W homologs of these species split soon after the divergence from the henophidian homologs (Figs. 3b and 4b; Additional files 9b and 10b). These suggest that the differentiation of Z and W homologs of the two genes began in an early caenophidian lineage after the divergence from henophidians. However, the phylogenetic placement of acrochordid Z and W homologs remains uncertain. Some trees clustered the acrochordid Z homolog with henophidian homologs (Fig. 3b and Additional file 10b) and others clustered it with the acrochordid W homolog (Fig. 4b), although an NJ tree (Additional file 9b) pointed to the affinity of the acrochordid Z and W homologs to Z and W homologs of other caenophidians, respectively. However, all these variations lack strong bootstrap supports and are thus unreliable. Taken together, our molecular phylogeny consistently supports the notion that differentiation between Z and W chromosomes began in an ancestral caenophidian lineage, although further data are required to resolve the phylogenetic placement of the acrochordid homologs.

### Heterochromatinization of W chromosomes in caenophidian snakes

**Fig. 6:**
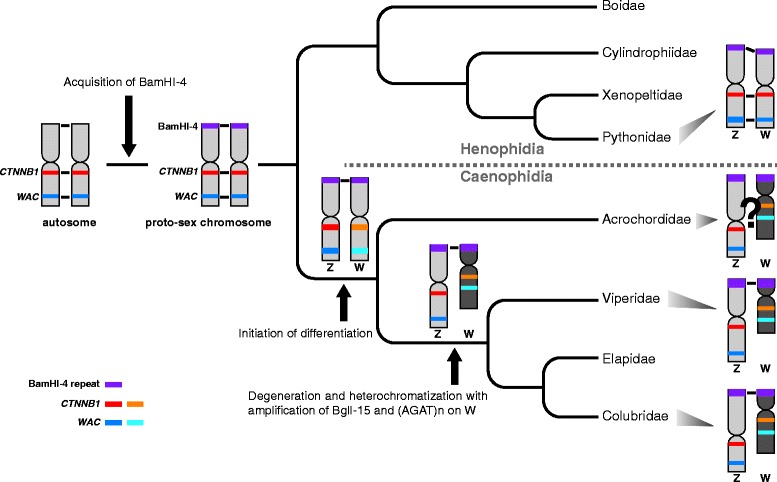
Evolution of snake sex chromosomes. The timing of evolutionary events on snake sex chromosomes inferred by this study is shown on the cladogram [49, 63]. Horizontal lines between Z and W chromosomes stand for the presence of recombination between the homologs on the chromosomes. Chromosome region with dark gray color stand for amplification of EQU-BglI-15 and (AGAT)_n_ repeats on the W chromosomes in caenophidian species. Note that morphologies of Z and W chromosomes and locations of the EQU-BamHI-4 repeat, *CTNNB1* and *WAC* genes in acrochordid species are not yet identified and that chromosomal locations of the two genes are also not yet identified in viperid species

The nucleotide sequence and chromosomal location of the EQU-BamHI-4 repetitive sequence are highly conserved in both henophidian and caenophidian species regardless of the degeneration status of their W chromosomes (Fig. 4 in [12], Fig. 5a–c). Therefore, this repetitive sequence was probably accumulated in the telomeric regions of short arms of sex chromosomes in the common ancestor of Henophidia and Caenophidia (Fig. 6).

Extensive amplification of the EQU-BglI-15 repetitive sequence was identified on the long arm of W chromosomes in the examined colubrid and viperid species, although an exception was found in *R. tigrinus* (Fig. 5d–f, Additional file 11a, b). Amplification of the (AGAT)_8_ microsatellite repeat motif was also identified on the long arm of W chromosomes in all the examined colubrid and viperid species (Fig. 5g–i and Additional file 11c). Although the chromosomal distribution of the two repeats in acrochordid and elapid species was not examined in this study, amplification of the (AGAT)_n_ motif was identified in several colubrid and elapid species [30–33, 57]. The amplification of repetitive sequences has frequently been associated with heterochromatinization of Y and W chromosomes in numerous animal and plant species (e.g., [28, 29, 57–59]). As nearly all colubrid, elapid, and viperid species have heterochromatic W chromosomes [12, 30, 57, 60, 61], our results suggest that heterochromatinization with accumulation of the EQU-BglI-15 and (AGAT)n sequences occurred on the W chromosomes in the common ancestor of Colubridae, Elapidae, and Viperidae (Fig. 6). It was recently reported that the dragonsnake (*Xenodermus javanicus*) from Xenodermatidae, another basally diverged lineage in the Caenophidia (Fig. 1), has morphologically differentiated Z and W chromosomes, and the amplification of (GATA)_n_ was identified on the highly heterochromatic W chromosome [62]. Thus, heterochromatinization with accumulation of the (AGAT)_n_ sequence probably occurred before the divergence of Xenodermatidae from other caenophidian families. The EQU-BglI-15 sequence does not contain the (AGAT)_n_ repeat in its sequence. Because these two repeats showed differential distributions on the W chromosomes in the four caenophidian species (Fig. 5d–i and Additional file 11), the two repeats could have spread on the caenophidian W chromosomes independently.

### Differentiation process of sex chromosomes in snakes

A schematic model for the differentiation process of sex chromosomes in snakes is shown in Fig. 6. At first, the EQU-BamHI-4 repetitive sequence was accumulated in the terminal regions of the short arms of Z and W chromosomes in the common ancestor of henophidians and caenophidians. The sex chromosomes of henophidian species have retained this little differentiated status to date. The size and morphology of Z chromosomes and gene orders of Z-linked genes are similar between the henophidian *P. bivittatus* and two caenophidian species, *E. quadrivirgata* and *P. flavoviridis* [12], suggesting that snake Z chromosomes have been conserved without large chromosomal rearrangements. In contrast, the W chromosomes of the two caenophidian species have reached a highly differentiated status. Although it is not yet clear whether the Z and W chromosomes are morphologically differentiated in acrochordid species [52, 53], our molecular phylogenetic data suggest that the cessation of recombination between the Z and W homologs of two genes (*CTNNB1* and *WAC*) that map on the centromeric and telomeric regions, respectively, of the long arms, began in the early stage of caenophidian divergence (Fig. 6). According to the predicted divergence times between snake families [63], differentiation of the proto-Z and proto-W chromosomes may have initiated and expanded to wide regions on the sex chromosomes in the common ancestor of caenophidian families during a relatively short period, 80–100 MYA (Fig. 1). A recent study using quantitative PCR (qPCR) on six putative sex chromosome-linked genes in 37 snake species reached a similar conclusion [64]. The qPCR analyses showed that female-to-male relative gene doses of the six genes were approximately 0.5 in all examined caenophidian species, including *A. granulatus* and *X. javanicus*, suggesting that the emergence of differentiated sex chromosomes preceded the diversification of caenophidian snakes [64]. The short arm of W chromosomes is extensively degenerated and, to the best of our knowledge, no gametologous gene has been cytogenetically identified there in caenophidian species. It is thus interesting to hypothesize that the degeneration and differentiation processes started earlier on the short arm than on the long arm, although more data is needed to verify this hypothesis.

## Conclusions

We studied the differentiation process of snake sex chromosomes using both coding sequences and repetitive sequences. We analyzed the molecular phylogeny of two gametologous genes, *CTNNB1* and *WAC*, and chromosomal distributions of sex chromosome-linked repetitive sequences in several snake species. Our results suggest that the differentiation between the proto-Z and proto-W chromosomes and heterochromatinization of the proto-W chromosome began in ancestral caenophidian lineages after their divergence from henophidians. However, these results were obtained using only a handful of genes and repetitive elements, and many details of the differentiation process (e.g., where on the W chromosomes the differentiation process initiated, and in which direction it proceeded) are still in question. In this regard, the present study provides solid progress in methodologies and phylogenetic information for further investigation of gametologs in snakes. In the future, genomic and cytogenetic approaches will be accelerated and provide critical information to elucidate the molecular mechanisms of sex chromosome evolution in vertebrates, including snakes.

## Additional files

Additional file 1: Primers used for this study. This table shows information of primers used for PCR amplification of partial sequences of *CTNNB1* and *WAC* genes, and for RACE to determine full-length cDNA sequences of the two genes in *E. quadrivirgata*. (XLSX 32 kb) Additional file 2: Diagrams for partial gene structure, primer positions and gel electrophoresis of PCR products. This file provides information about primer positions on *CTNNB1* and *WAC* genes, and images of gel electrophoreses for PCR amplicons of partial sequences of *CTNNB1* gene in snakes. (PDF 401 kb) Additional file 3: Accession numbers of snake contigs that contain genomic sequences of *CTNNB1* and *WAC* genes. This table shows accession numbers of contigs containing genomic sequences of *CTNNB1* and *WAC* genes of four snake species, *P. bivittatus*, *O. hannah*, *C. mitchellii* and *V. berus*. (XLSX 33 kb) Additional file 4: Models and parameters used for estimation of molecular phylogenetic trees. This table shows models and parameters used for estimation of molecular phylogenetic trees. (XLSX 44 kb) Additional file 5: Accession numbers of *CTNNB1* and *WAC* gene sequences for snake species determined in this study. This table shows accession numbers of partial or full-length *CTNNB1* and *WAC* gene sequences for snake species determined in this study. (XLSX 35 kb) Additional file 6: Pairwise sequence similarities of *CTNNB1* and *WAC* genes among nine tetrapods. This table shows pairwise sequence similarities of *CTNNB1* and *WAC* genes among nine tetrapods. (XLSX 39 kb) Additional file 7: Alignment of full-length amino acid sequences of CTNNB1 among tetrapod species. This figure shows alignment of full-length amino acid sequences of CTNNB1 among 11 tetrapod species. (PDF 306 kb) Additional file 8: Alignment of full-length amino acid sequences of WAC among tetrapod species. This figure shows alignment of full-length amino acid sequences of WAC among 11 tetrapod species. (PDF 302 kb) Additional file 9: Molecular phylogenic trees of *CTNNB1* gene. This figure shows neighbor-joining trees of *CTNNB1* gene with the long alignment for 20 tetrapod species and the short alignment for 26 squamate species. (PDF 289 kb) Additional file 10: Molecular phylogenic trees of *WAC* gene. This figure shows neighbor-joining trees of *WAC* gene with the long alignment for 21 tetrapod species and the short alignment for 21 squamate species. (PDF 282 kb) Additional file 11: FISH of EQU-BglI-15 repetitive sequence and (AGAT)_8_ microsatellite motif in *E. quadrivirgata* and *G. blomhoffii*. This figure shows FISH images of EQU-BglI-15 repetitive sequence and (AGAT)_8_ microsatellite motif in *E. quadrivirgata* and *G. blomhoffii*. (PDF 814 kb)

## Abbreviations

*CTNNB1*: Catenin (cadherin-associated protein), beta 1, 88 kDa
dUTP: Deoxyuridine triphosphate
EBI: The European bioinformatics institute
EMBL: European molecular biology laboratory
EST: Expressed sequence tag
FISH: Fluorescence *in situ* hybridization
INSDC: International nucleotide sequence database collaboration
NCBI: National center for biotechnology information
ORF: Open reading frame
PCR: Polymerase chain reaction
PI: Propidium iodide
RACE: Rapid amplification of cDNA ends
UTR: Untranslated region
*WAC*: WW domain containing adaptor with coiled-coil
