# Supplementary material for: Sex chromosome evolution in snakes inferred from divergence patterns of two gametologous genes and chromosome distribution of sex chromosome-linked repetitive sequences
Source: Zoological Lett. 2016 Aug 26;2(1):19. doi: 10.1186/s40851-016-0056-1 (PMC5002183; doi:10.1186/s40851-016-0056-1)
Supplement: Additional file 2: — Diagrams for partial gene structure, primer positions and gel electrophoresis of PCR products. This file provides information about primer positions on CTNNB1 and WAC genes, and images of gel electrophoreses for PCR amplicons of partial sequences of CTNNB1 gene in snakes. (PDF 401 kb) [file 40851_2016_56_MOESM2_ESM.pdf]

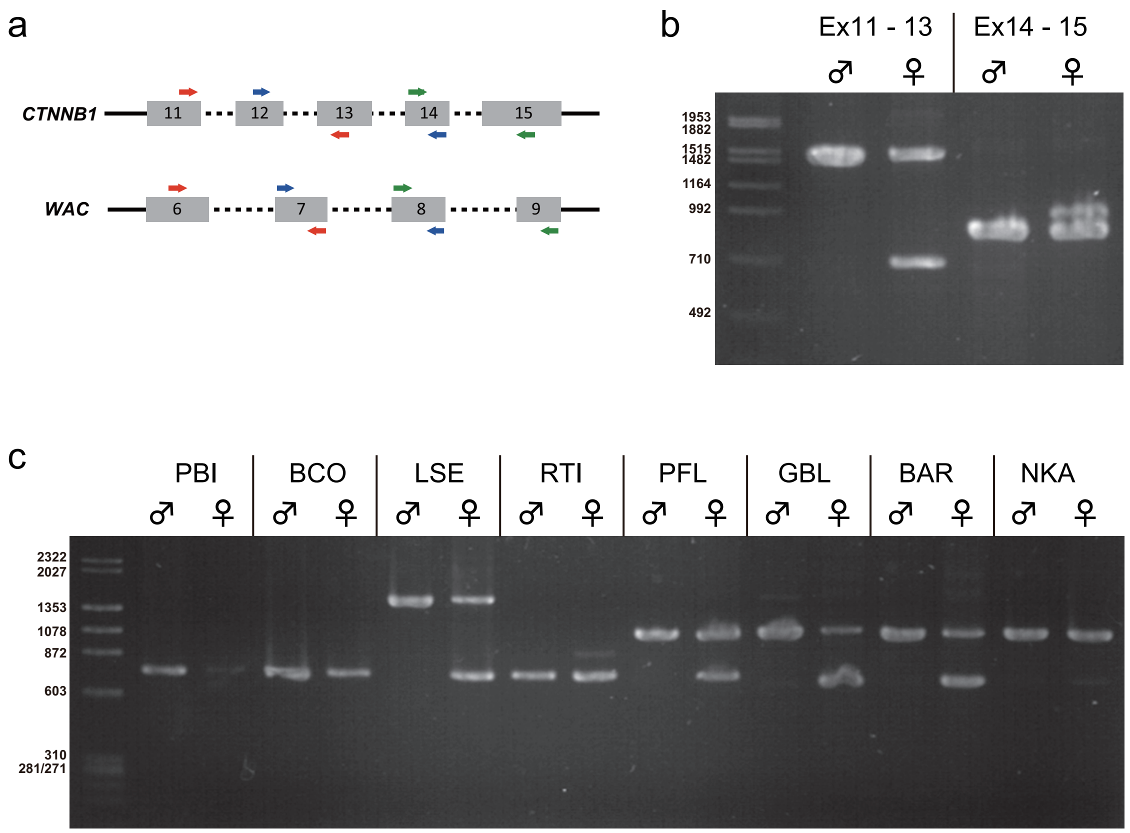

Additional file 2. Diagrams for partial gene structure, primer positions and gel electrophoresis of PCR products. a. Diagrams show partial structures of *CTNNB1* and *WAC* genes inferred from human, chicken and green anole homologs with positions of primers used for PCR. Solid and dashed lines show introns, and grey boxes show exons. Colored arrows show positions of three primer pairs. **b.** Gel electrophoresis image is shown for PCR products amplified with two primer pairs, Eq-CTNNB1-11-F × -13-R and Eq-CTNNB1-14-F × -15-R, in male and female *E. quadrivirgata*. **c.** Gel electrophoresis image is shown for PCR products amplified with a primer pair, Eq-CTNNB1-11-F × -13-R, in males and females of *P. bivittatus* (PBI), *B. constrictor* (BCO), *L. semicarinatum* (LSE), *R. tigrinus* (RTI), *P. flavoviridis* (PFL), *G. blomhoffii* (GBL), *B. arietans* (BAR), and *N. kaouthia* (NKA).
