# Supplementary material for: Sex chromosome evolution in snakes inferred from divergence patterns of two gametologous genes and chromosome distribution of sex chromosome-linked repetitive sequences
Source: Zoological Lett. 2016 Aug 26;2(1):19. doi: 10.1186/s40851-016-0056-1 (PMC5002183; doi:10.1186/s40851-016-0056-1)
Supplement: Additional file 8: — Alignment of full-length amino acid sequences of WAC among tetrapod species. This figure shows alignment of full-length amino acid sequences of WAC among 11 tetrapod species. (PDF 302 kb) [file 40851_2016_56_MOESM8_ESM.pdf]

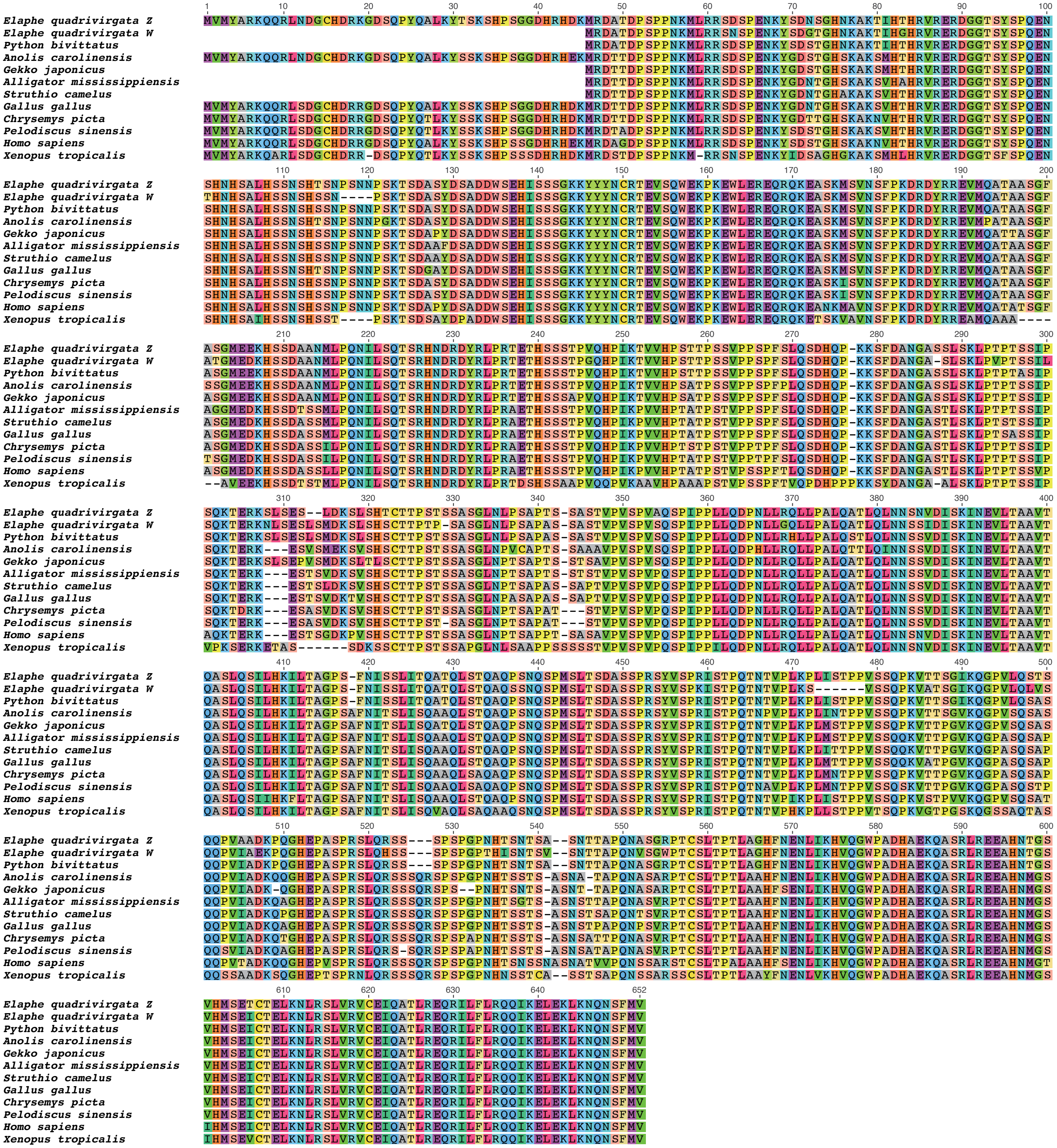

Additional file 8. Alignment of full-length amino acid sequences of WAC among tetrapod species. Predicted WAC amino acid sequences of *E. quadrivirgata* Z and W homologs are aligned with the homologs of 10 tetrapods species, *P. bivittatus*, *A. carolinensis*, *G. japonicus*, *A. mississippiensis*, *S. camelus*, *G. gallus*, *C. picta*, *P. sinensis*, *H. sapiens*, and *X. tropicalis*. An initial part of the *E. quadrivirgata* W homolog was trimmed as a large deletion that would cause a frameshift was identified in the regions (see text and Fig. 2 for detailed information).
