# Supplementary material for: Sex chromosome evolution in snakes inferred from divergence patterns of two gametologous genes and chromosome distribution of sex chromosome-linked repetitive sequences
Source: Zoological Lett. 2016 Aug 26;2(1):19. doi: 10.1186/s40851-016-0056-1 (PMC5002183; doi:10.1186/s40851-016-0056-1)
Supplement: Additional file 9: — Molecular phylogenic trees of CTNNB1 gene. This figure shows neighbor-joining trees of CTNNB1 gene with the long alignment for 20 tetrapod species and the short alignment for 26 squamate species. (PDF 289 kb) [file 40851_2016_56_MOESM9_ESM.pdf]

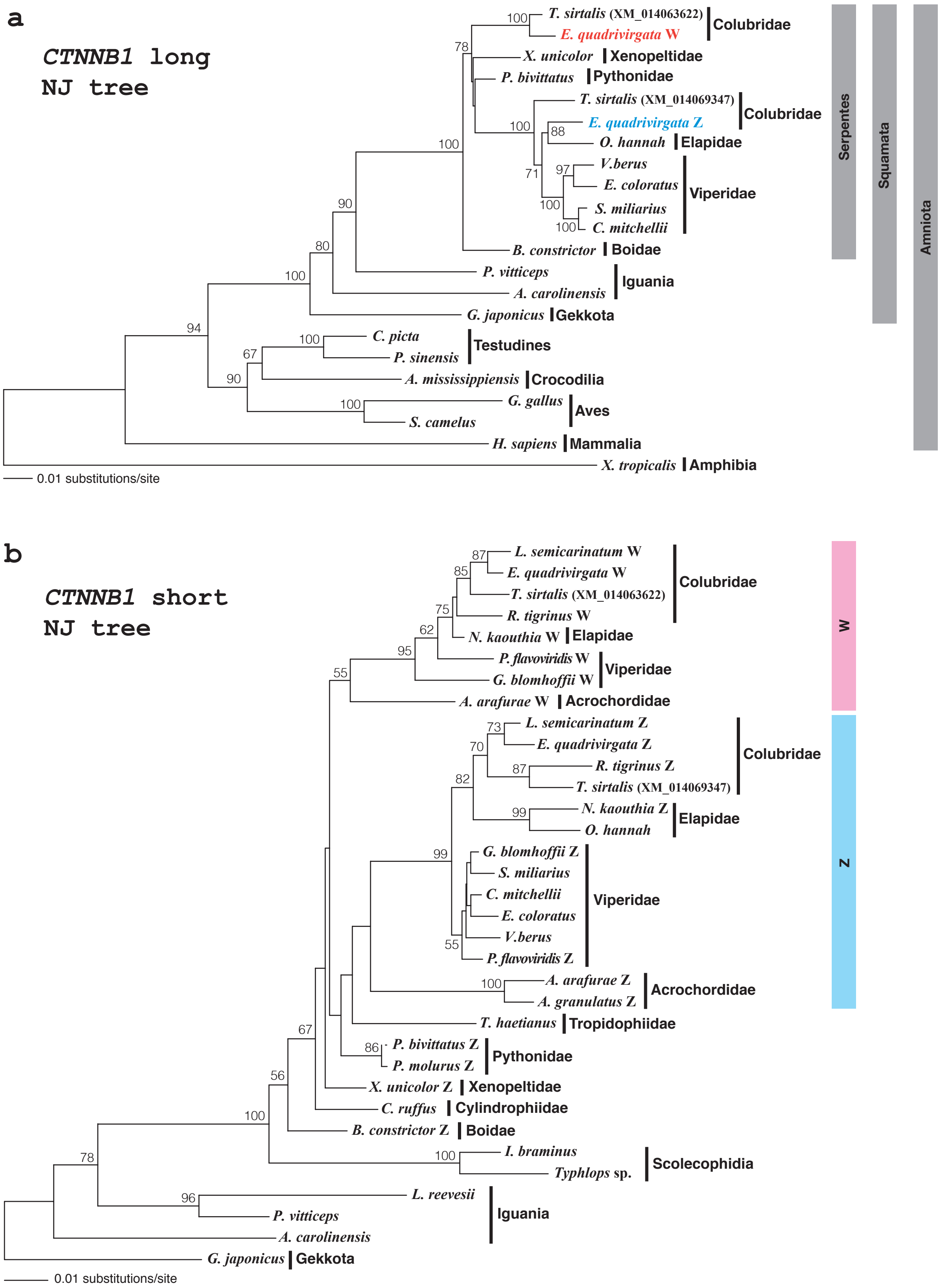

Additional file 9. Molecular phylogenic trees of *CTNNB1* gene. Neighbor-joining (NJ) trees of *CTNNB1* genes were constructed with the long alignment for 20 tetrapod species **(a)** and the short alignment for 26 squamates species **(b)**. Bootstrap values (>50%) are shown on each node. Classification is shown on the right side of species. Blue and pink bars in **b** show clades of Z and W homologs of caenophidian species.
