# Supplementary material for: Sex chromosome evolution in snakes inferred from divergence patterns of two gametologous genes and chromosome distribution of sex chromosome-linked repetitive sequences
Source: Zoological Lett. 2016 Aug 26;2(1):19. doi: 10.1186/s40851-016-0056-1 (PMC5002183; doi:10.1186/s40851-016-0056-1)
Supplement: Additional file 11: — FISH of EQU-BglI-15 repetitive sequence and (AGAT)8 microsatellite motif in E. quadrivirgata and G. blomhoffii. This figure shows FISH images of EQU-BglI-15 repetitive sequence and (AGAT)8 microsatellite motif in E. quadrivirgata and G. blomhoffii. (PDF 814 kb) [file 40851_2016_56_MOESM11_ESM.pdf]

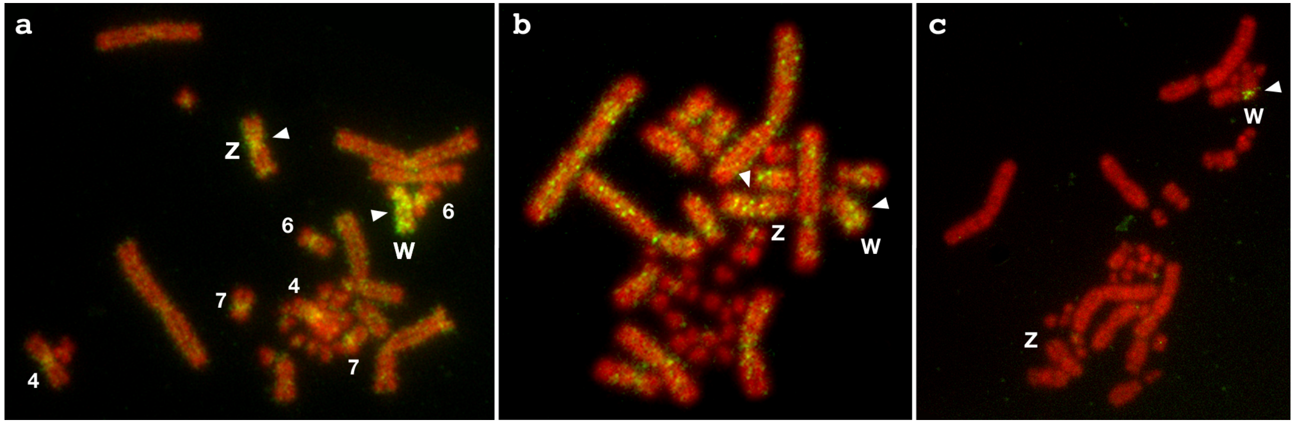

Additional file 11. FISH of EQU-BglI-15 repetitive sequence and $(AGAT)_8$ microsatellite motif in *E. quadrivirgata* and *G. blomhoffii*. FITC-labelled *E. quadrivirgata* BglI-15 repeat was hybridized to PI-stained metaphase spreads of *E. quadrivirgata* **(a)** and *G. blomhoffii* **(b)**. The $(AGAT)_8$ microsatellite motif was hybridized to metaphase spreads of *G. blomhoffii* **(c)**. Arrowheads indicate hybridization signals on sex chromosomes.
